# Toolbox approach for fast generation of stable CHO production cell lines from different hosts

**DOI:** 10.1186/1753-6561-5-S8-P28

**Published:** 2011-11-22

**Authors:** Susanne Seitz, Henning von Horsten, Thomas Rose, Volker Sandig, Karsten Winkler

**Affiliations:** 1 ProBioGen AG, Berlin, 13086, Germany

## Background

Protein production in CHO cell lines is a highly dynamic process, where the yield limiting step can shift among transcription, translation, and secretion under different cell growth stages and culture conditions. In addition, the choice of CHO cell line has been shown to affect target protein quality and quantity.

Further advancements in titer and specific productivity require elimination of cellular/process bottlenecks along the generation of stable production cell lines including vector design, clone selection, genetic engineering, and media and feed optimization.

To overcome these limitations, we developed a toolbox for all process phases from transfection to production. This toolbox is based on a chemically defined media platform and includes pre-adapted CHO-K1 or CHO-DG44 cell lines, optimized vectors for single and multi-chain proteins as well as fine-tuned protocols. In addition, it enables the production of antibodies with specialized glycan structures (GlymaxX® [1]) as well as clone engineering with regard to the expression and secretion machinery (CDC42).

## Materials and methods

ProBioGen‘s toolbox expression vectors encoding an Fc-protein or therapeutic glycoprotein were generated using gene-optimized sequences and optimized signal peptides.

To create stable cell lines, expression vectors were transfected into CHO-DG44 and CHO-K1 cells by microporation. Two serum-free selection strategies were applied using both MTX and puromycin: selection in semi-solid medium followed by automated clone picking and deposition into 96 wells using the ClonePix FL (Genetix), and combined selection and cloning in 96-well plates. For a first expression analysis monoclonal primary clones were subjected to 96-well plate assay. Clones showing best performance in 96-well were expanded and taken into batch and fed-batch for productivity assessment. Best performing primary clones were subjected to a second cloning step by limiting dilution and analyzed in 96-well. Following clone scale-up highest ranking sub-clones clones were assessed in shake flask using fed-batch. Prior to master cell banking candidate lead clones were tested for fed-batch bioreactor performance in a Cellferm-pro® (DASGIP AG). Protein concentrations were determined using either an ELISA method, the Gyrolab SIA Ligand Binding Assay or an HPLC method.

Protein secreted from stable cell lines was purified either by protein A affinity chromatography (Fc-protein) or Ion Exchange Chromatography using weak anion exchangers (glycoprotein) and evaluated by Size-Exclusion HPLC (SEC), Western blot and SDS-PAGE.

To determine the stability of clones a continuous passage of roughly 80 population doublings with analyzing protein batch titers as well as mRNA levels and gene copy numbers (StepOnePlus™ RT-PCR System) at defined time points was performed. Cells were cultivated without selection pressure.

## Results and conclusion

**Figure 1:**
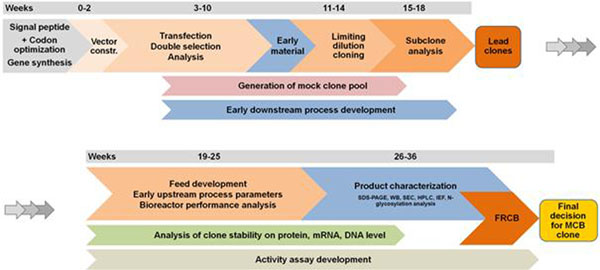
Overview of PBG Cell Line Development

Here we describe an innovative parallel platform cell line development for CHO-K1 and CHO-DG44 (Figure 1) exemplified by two completely different recombinant proteins, an Fc-protein and a therapeutic glycoprotein, with the glycoprotein showing differences in glycosylation pattern in the two starter cell lines.

Our toolbox approach enables a fast and highly reproducible generation of high producer clones stably expressing the transgene for more than 80 generations without selection pressure and resulting in upstream yields of 4.5-13 g/L (Table 1).

**Table 1:** Growth and protein production data of CHO producer clones in different assay formats

		Fc-protein		Glycoprotein	
**Assay format**	**Cell line**	**Max VCD [viable cells/mL]**	**Max Titer [mg/L]**	**Max VCD [viable cells/mL]**	**Max Titer [mg/L]**

**96-well plate assay**	K1	nd^a^	50	nd^a^	268
	
**(5 days)**	DG44	nd^a^	176	nd^a^	607

**Batch**	K1	2.3E+07	610	1.1E+07	1772
	
**(5 or 7 days)**	DG44	8.6E+06	980	6.4E+06	2200

**Fed batch**	K1	3.0E+07	2500	1.9E+07	8100
	
**(12-17 days)**	DG44	1.7E+07	4500	3.0E+07	13200

^a^ not determined
